# Variation in meningioma recurrence risk estimates across observational cohorts: the influence of calendar time, WHO classifications, geographical settings, and healthcare systems

**DOI:** 10.1007/s11060-026-05753-7

**Published:** 2026-08-28

**Authors:** Christian Mirian, Lasse Rehné Jensen, Adam Gorm Hoffmann, Tareq A. Juratli, Anders Broechner, Sverre H. Torp, Helen A. Shih, Ramin A. Morshed, Jacob S. Young, Stephen T. Magill, Luca Bertero, Walter Stummer, Dorothee Cäcilia Spille, Benjamin Brokinkel, Soichi Oya, Satoru Miyawaki, Nobuhito Saito, Martin Proescholdt, Yasuhiro Kuroi, Konstantinos Gousias, Matthias Simon, Jennifer Moliterno, Ricardo Prat-Acin, Stéphane Goutagny, Vikram C. Prabhu, John T. Tsiang, Johannes Wach, Erdem Güresir, Junkoh Yamamoto, Young Zoon Kim, Joo Ho Lee, Daniel W. Kim, Matthew Koshy, Karthikeyan Perumal, Mustafa K. Baskaya, Donald M. Cannon, Dennis C. Shrieve, Chang-Ok Suh, Jong Hee Chang, Maria Kamenova, Sven Straumann, Jehuda Soleman, Ilker Y. Eyüpoglu, Tony Catalan, Austin Lui, Philip V. Theodosopoulos, Michael W. McDermott, Fang Wang, Pedro Góes, Manoel Antonio de Paiva Neto, Ricardo Komotar, Michael E. Ivan, Aria Jamshidi, Evan Luther, Luis Souhami, Marie-Christine Guiot, Tamás Csonka, Toshiki Endo, Olivia Claire Barrett, Randy Jensen, Tejpal Gupta, Akash J. Patel, Tiemo J. Klisch, Jun Won Kim, Francesco Maiuri, Valeria Barresi, María Dolores Tabernero, Simon Skyrman, Ian Law, Bjarne Winther Kristensen, Tina Nørgaard Munch, Torstein Meling, Kåre Fugleholm, Paul Blanche, Tiit Mathiesen, Andrea Daniela Maier

**Affiliations:** 1 Department of Neurosurgery, Copenhagen University Hospital, Blegdamsvej 9, Copenhagen, 2100 Denmark; 2 Section of Biostatistics, Department of Public Health, University of Copenhagen, Copenhagen, Denmark; 3 Department of Neurosurgery, Division of Neuro-Oncology, Faculty of Medicine, University Hospital Carl Gustav Carus, Technische Universität Dresden, 01307 Dresden, Germany; 4 Department of Neurosurgery, Laboratory of Translational Neuro-Oncology, Massachusetts General Hospital Cancer Center, Harvard Medical School, Boston, USA; 5 Department of Clinical and Molecular Medicine, Faculty of Medicine and Health Sciences, Laboratory Centre, Norwegian University of Science and Technology (NTNU), St. Olavs Hospital, Trondheim, NO-7491 Norway; 6 Department of Pathology, Laboratory Centre, St. Olavs hospital, Trondheim, NO-7030 Norway; 7 Department of Radiation Oncology, Harvard Medical School, Massachusetts General Hospital, Mass General Brigham Cancer Institute, Boston, MA USA; 8 Department of Neurological Surgery, University of California San Francisco, San Francisco, CA USA; 9 Department of Neurological Surgery, Feinberg School of Medicine, Northwestern University, Evanston, IL USA; 10 Pathology Unit, Department of Medical Sciences, University and Città della Salute e della Scienza University Hospital of Turin, Turin, Italy; 11 Department of Neurosurgery, University of Münster, Münster, Germany; 12 Institute for Neuropathology, University of Münster, Münster, Germany; 13 Deparment of Neurosurgery, Clemenshospital Münster, Münster, Germany; 14 Department of Neurosurgery, Saitama Medical Center/University, Saitama, Japan; 15 Department of Neurosurgery, The University of Tokyo Hospital, Tokyo, Japan; 16 Department of Neurosurgery, University Regensburg Medical Center, Regensburg, Germany; 17 Department of Neurosurgery, Tokyo Women’s Medical University, Adachi Medical Center, Tokyo, Japan; 18 Department of Neurosurgery, Athens Medical Center, Athens, Greece; 19 EUC Athens Medical School, Athens, Greece; 20 Department of Neurosurgery, Bethel Clinic University of Bielefeld Medical Center, Bielefeld, Germany; 21 Department of Neurosurgery, Yale School of Medicine Yale New Haven Hospital, Smilow Cancer Hospital, New Haven, USA; 22 Department of Neurosurgery, Hospital La Fe, Valencia, Spain; 23 Université Paris Cité, Department of Neurosurgery, Beaujon Hospital, Assistance Publique Hôpitaux de Paris, Paris, France; 24 Department of Neurological Surgery, Loyola University Medical Center, Stritch School of Medicine, Maywood, IL USA; 25 Department of Neurosurgery, University Hospital Leipzig, Leipzig, Germany; 26 Department of Neurosurgery, University of Occupational and Environmental Health, Kitakyushu, Japan; 27 Department of Neurosurgery, Samsung Changwon Hospital, Sungkyunkwan University School of Medicine, Changwon, Republic of Korea; 28 Department of Radiation Oncology, Seoul National University College of Medicine, Seoul National University Hospital, Seoul, Republic of Korea; 29 Cancer Research Institute, Seoul National University College of Medicine, Seoul, South Korea; 30 Department of Radiation Oncology, University of Illinois Hospital and Health Sciences System, Chicago, IL USA; 31 Department of Neurosurgery, University of Wisconsin Medical School & Public Health, Madison, WI USA; 32 Department of Radiation Oncology Spencer Fox Eccles School of Medicine, University of Utah, Salt Lake City, UT USA; 33 Department of Radiation Oncology, Yonsei University College of Medicine, Seoul, Republic of Korea; 34 Department of Neurosurgery, Yonsei University College of Medicine, Seoul, Republic of Korea; 35 Department of Neurosurgery, University Hospital Basel, Basel, Switzerland; 36 Division of Neurosurgery, Miami Neuroscience Institute, Miami, FL USA; 37 Department of Neurosurgery, The First Affiliated Hospital of Zhengzhou University, Zhengzhou, Henan China; 38 Department of Neurosurgery, Federal University of São Paulo, São Paulo, Brazil; 39 Department of Neurological Surgery, Sylvester Comprehensive Cancer Center, University of Miami, Coral Gables, FL USA; 40 Department of Neurosurgery, Hackensack Meridian Health, Edison, NJ USA; 41 Department of Neurosurgery, Allegheny General Hospital, Pittsburgh, PA USA; 42 Division of Radiation Oncology, McGill University Health Centre, McGill University, Montreal, Quebec, Canada; 43 Department of Pathology, McGill University Health Centre, Montreal, Quebec, Canada; 44 Department of Pathology, Faculty of Medicine, University of Debrecen, Debrecen, Hungary; 45 Division of Neurosurgery, Tohoku Medical and Pharmaceutical University, Tohoku, Japan; 46 Infirmary Cancer Care, Mobile, AL USA; 47 Department of Neurosurgery, Huntsman Cancer Institute, University of Utah, Salt Lake City, UT USA; 48 Department of Radiation Oncology ACTREC, Tata Memorial Centre, HBNI Kharghar, Navi Mumbai, 410210 India; 49 Department of Neurosurgery, Baylor College of Medicine, Houston, TX USA; 50 Department of Otolaryngology-Head and Neck Surgery, Baylor College of Medicine, Houston, TX USA; 51 Duncan Neurological Research Institute, Texas Children’s Hospital, Houston, TX USA; 52 Department of Molecular and Human Genetics, Baylor College of Medicine, Houston, TX USA; 53 Department of Radiation Oncology, Gangnam Severance Hospital, Yonsei University College of Medicine, Seoul, Republic of Korea; 54 Department of Neurosurgery, University of Naples Federico II, Naples, Italy; 55 Department of Diagnostics and Public Health, University of Verona, Verona, Italy; 56 Unit of Anatomic Pathology- Neuropathology, Fondazione IRCCS Istituto Neurologico Carlo Besta, Milan, Italy; 57 Instituto de Investigación Biomédica de Salamanca (IBSAL), University Hospital of Salamanca, Salamanca, Spain; 58 Department of Clinical Neuroscience, Karolinska Institutet, Stockholm, Sweden; 59 Department of Clinical Physiology and Nuclear Medicine, Copenhagen University Hospital-Rigshospitalet, Copenhagen, Denmark; 60 Department of Clinical Medicine, Faculty of Health and Medical Sciences, University of Copenhagen, Copenhagen, Denmark; 61 Department of Clinical Medicine and Biotech Research and Innovation Center (BRIC), University of Copenhagen, Copenhagen, Denmark; 62 Department of Pathology, Bartholin Institute, Rigshospitalet, Copenhagen University Hospital, Copenhagen, Denmark; 63 Department of Congenital Disorders, Statens Serum Institut, Copenhagen, Denmark; 64 Department of Neurological Surgery, Istituto Nazionale Neurologico “C.Besta”, Milan, Italy

**Keywords:** Meningioma, Neuro-oncology, Epidemiology, Recurrence

## Abstract

**Purpose:**

Recurrence risk estimates underpin meningioma research, including molecular classification and clinical trial benchmarking, yet are often based on retrospective or historical data. The aim of this study was to assess the variation of recurrence risk estimates across calendar periods, WHO classification editions, geographical settings, and healthcare systems.thetermine

**Methods:**

We analyzed 4,111 patients with primary WHO-1/-2 meningiomas from 31 centers in 15 countries (1990–2019). Recurrence was defined according to local radiological assessment. The 5- and 10-year recurrence risks were estimated using regression standardization with inverse probability of censoring weights, adjusting for key clinical, surgical, and histopathological variables.

**Results:**

Recurrence risk estimates varied across all domains examined. More recent calendar periods were associated with higher predicted recurrence risk, particularly for WHO-2 at 5 years (e.g., ≥ 2013 vs. ≤ 2007: RR 1.60, 95% CI 1.19–2.01). Differences were also observed across healthcare systems and geographical settings despite adjustment. In contrast, recurrence risk estimates were broadly comparable between WHO classification editions (2007 vs. 2016). Cohort-level visualization revealed substantial heterogeneity in follow-up duration, inclusion patterns, and recurrence detection.

**Conclusions:**

Recurrence risk estimates are not stable across time or settings and are influenced by several patterns, particularly data accrual patterns. Recurrence events are inherently “locked” to the historical context in which they were detected, which may have important implications for developing molecular classifiers using retrospective specimens and clinical trials relying on benchmark established in historical settings. We propose transparent visualization of observational cohort composition and individual-level event timing to improve interpretability and comparability.

**Supplementary Information:**

The online version contains supplementary material available at https://doi.org/10.1007/s11060-026-05753-7.

## Introduction

Many single-center meningioma cohorts extend over several years or even decades, reflecting the long follow-up required to study recurrence risks given the slow-growing nature of most tumors [1–5]. A common approach in such studies is to compare recurrence risk estimates with historical cohorts that appear similar in design; however, the lack of individual-level data to adjust for confounders and biases limits these comparisons to qualitative rather than quantitative evaluation. The variation of recurrence risk estimates across calendar time, WHO classification editions, and healthcare settings has not been investigated, although clinical practice, diagnostic tools, and various types of classification systems are introduced and refined over time [6–18]. Historically, recurrence was defined by Donald Simpson as the “reappearance of symptoms due directly to tumour growth after a period of symptomatic relief”, while contemporary imaging approaches, such as high-resolution [^68^Ga]Ga-DOTA-TOC PET, can detect meningioma lesions as small as 0.1 cm^3^ [19, 20]. Consequently, comparing recurrence risks across extended periods, may be misleading because changes in detection methods, including advances in technology and the lack of standardized follow-up protocols, as well as evolving definitions such as revisions to the WHO classification, inevitably influence the results. In most observational cohorts, recurrence reflects detection in routine clinical practice rather than a uniformly defined radiological endpoint. As such, reported recurrence risk estimates inherently depend on surveillance intensity, imaging modalities, and diagnostic thresholds; however, the extent of this impact on recurrence risk estimates across time, geographical, and healthcare settings remains unclear [6, 13, 15–17, 21, 22].

Our primary aim was to explore how recurrence risk estimates may vary across calendar time, WHO classification editions, geographical settings, and healthcare systems by analysing individual-level data from 4,111 primary meningioma patients diagnosed and followed between 1990 and 2019 across 31 centers in 15 countries. Specifically, we investigated whether recurrence risk estimates differed when standardized to the same distribution of patient, tumor, and treatment characteristics but evaluated under different calendar periods, classification editions, geographical settings, and healthcare systems. Thus, this study did not aim to identify prognostic determinants of recurrence, but rather to assess the variation of recurrence risk estimates across observational settings.

## Materials and methods

### The *PERNS* database

The *PERNS* database (PERsonalized NeuroSurgery) is an international retrospective collaborative platform established to harmonize individual-level clinical, surgical, histopathological, and follow-up data from patients with primary meningiomas diagnosed, treated, and followed between 1990 and 2019. Participating centers were included based on their willingness and ability to contribute retrospective datasets. The database was not designed as a population-based registry and therefore does not aim to capture all meningioma cases treated within participating regions or healthcare systems. Missing data primarily reflect differences in variable availability and recording practices between centers.

### Study population

Patients included were classified according to either the 2007 or 2016 WHO classification edition, had a primary WHO-1 or WHO-2 meningioma, and no missing information on the variables described below. WHO classification reflected the classification recorded within the original contributing datasets, including retrospective reclassification of historical cases in centers where such reviews had been performed. All individuals were 18.0 years or older at the time of surgery. A total of 235 (5.7%) patients received adjuvant fractionated radiotherapy (aFRT) after surgery. The study population consisted exclusively of patients with primary intracranial meningiomas. Patients were followed from the time of surgery until recurrence, recurrence-free death, or last clinical follow-up, whichever occurred first.

### Outcome, exposure, and comparisons

The 5- and 10-year postoperative risks of recurrence constitute the main endpoints. Recurrence was assessed locally based on radiological evaluation without standardized criteria, as most cases predated the introduction of the *Response Assessment in Neuro-Oncology* (RANO) framework [23]. Although evaluated in multidisciplinary teams, the outcome reflects recurrence as detected within heterogeneous follow-up practices. The main analysis was based on regression standardization (also known as *G*-computation), which estimates the difference in average risk of recurrence that would be expected between two patient groups if they had the exact same distribution of observed characteristics, differing only in the characteristic that defines the groups [24]. In this way, it isolates differences that cannot be explained by the variables included in the adjusted model. Remaining differences may still reflect unmeasured factors, including center-specific selection mechanisms, follow-up practices, or data accrual patterns. Patients were grouped and compared in four ways (further detailed below): (1) by calendar-time period at diagnosis, (2) by WHO classification edition, (3) by geographical setting, and (4) by type of national healthcare system.

### Definition of variables

Calendar time of diagnosis was categorized into three groups: ≤2007, 2008–2012, and ≥ 2013. The year 2007 was selected as cut-off because a WHO classification revision was published that year. A 2016 cut-off was not feasible, as no patients diagnosed then could have reached five years of follow-up (data accrual closed in 2019). The 2013 cut-off was chosen since this yielded approximately equal group sizes.

The following variables were also included in the regression models: age at surgery (categorically: <50, between 50 and 70, or > 70 years), sex, intracranial location (skull-base or non-skull base), WHO grade (WHO-1 or WHO-2), WHO classification (the 2007 or 2016 edition), the Ki-67 proliferation index (PI), Simpson grade (1–4, which was determined intraoperatively), whether aFRT was administered (yes or no), and the healthcare system setting, categorized as Tax-funded health services (Spain, Italy, Norway, Sweden, Canada), Social insurance-funded (mandatory multi-payer insurance; Germany, France, Switzerland, Hungary, Japan, South Korea), or Mixed/Private-funded with financing from private sector involvement (USA, India, China).

### Multivariable regression analysis

Regression standardization was performed using multiple logistic regression [24]. Separate logistic models were fitted using inverse probability of censoring weights (IPCW); one for each of the four comparisons [25]. The censoring weights were computed by using the Kaplan-Meier method, stratified by the individual cohorts and calendar time-group. All models were adjusted for the variables defined above, except for calendar time, WHO grade, WHO classification edition, geographical setting, and healthcare system, which were incorporated differently to reflect each of the four comparisons. In addition, an interaction term between Simpson grade and Ki-67 PI was included to account for a possibly different effect of Ki-67 PI on recurrence risk depending on extent of resection [26]. Recurrence-free death was considered a competing event [1].

### Regression standardization

All four multivariable logistic regression models were fitted using the full dataset. For each comparison, a standard population was selected, and the fitted models were used to predict the average risk of recurrence in that population for each of the patient groups under comparison. Specifically, the average recurrence risk was estimated by first predicting a recurrence risk estimate for each individual and then computing a weighted average of these predictions across the entire population (with weights reflecting the distribution of patient characteristics in the standard population) [24]. The 95% confidence intervals (CI) were calculated using a bootstrap resampling approach [25].

**Comparison 1: Calendar time-groups** Patients diagnosed between 2008 and 2012 were used as the standard population. Accordingly, we estimated differences in recurrence risk had they been diagnosed in a different calendar periods, i.e. standardized to the distribution of patient characteristics corresponded exactly to those observed in the patients diagnosed in 2008–2012. Thus, any estimated difference in recurrence risk should not be attributable to changes in the distribution of these patient characteristics between the calendar periods.

The analysis was stratified by WHO grade, and the model further incorporated calendar time-group, WHO classification edition, and healthcare system. Interaction terms between calendar time-group and WHO classification edition, and between calendar time-group and healthcare system, were included to account for how these factors may influence each other’s effect on recurrence risk estimates.

**Comparison 2: The WHO classification edition** Differences in recurrence risk estimates between the 2007 and 2016 WHO classification editions were estimated, using the patient characteristics of those diagnosed under the 2007 edition as the standard population. Hence, recurrence risks under both WHO editions were predicted for the patient characteristics observed in patients diagnosed according to the 2007 edition and averaged over this distribution. This attenuates the possibility that any observed difference in recurrence risk is not driven by differences in the distribution of included patient characteristics between the WHO classification groups. Two interaction terms were specified: one between WHO classification edition and calendar time-group, and another between WHO classification edition and healthcare system.

**Comparison 3: Geographical setting** Only European centers with Tax- or Social insurance-funded healthcare systems were included for this scenario. The analysis was further limited to cohorts with a median follow-up of at least 5.0 years and a minimum of 20 patients with WHO-2. A cohort from the Division of Neurosurgery, Geneva University Hospitals (Switzerland) was selected as the standard population, as this cohort included individuals across all covariate categories (Table 1). Seven centers met these criteria, and recurrence risk was estimated for each center standardized to the distribution of patient characteristics within each cohort matched exactly that of the Geneva cohort. The identities of the individual centers are not disclosed, as the purpose of this study was not to evaluate single-center performance; however, the center were in Central Europe (*n* = 2), Southern Europe (*n* = 3), South-Western Europe (*n* = 1), and Northern Europe (*n* = 1). The model included the individual centers as a covariate, which also included interaction terms between WHO grade and Ki-67 PI and between WHO grade and Simpson grade.

**Table 1:** Distribution of patient characteristics in the Geneva-cohort used as standard population and compared to the distribution of characteristics across the compiled patients

	Geneva-cohort *n* = 410	All other cohorts compiled *n* = 3,701
Age group: <50.0 years	107 (26%)	998 (27%)
50.0–70.0 years	223 (54%)	1,912 (52%)
> 70.0 years	80 (20%)	791 (21%)
Female sex	299 (73%)	2,595 (70%)
Male	111 (27%)	1,106 (30%)
WHO-1	360 (88%)	2,648 (72%)
WHO-2	50 (12%)	1,053 (28%)
Simpson Grade 1	152 (37%)	1,471 (40%)
Grade 2	165 (40%)	1,108 (30%)
Grade 3	32 (8%)	443 (12%)
Grade 4	61 (15%)	679 (18%)
Ki-67 Proliferation Index*	3.0 (1.0, 5.0)	3.0 (1.0, 5.0)
Skull-base Location	221 (54%)	1,982 (54%)
Non-Skull-base Location	189 (46%)	1,719 (46%)
No aFRT	410 (100%)	3,466 (94%)
Yes aFRT	0 (0%)	235 (6.3%)
Year of diagnosis (≤2007)	34 (8%)	1,534 (41%)
Between 2008–2012	172 (42%)	1,444 (39%)
≥2013	204 (50%)	723 (20%)

*Abbreviations.* aFRT: Adjuvant Fractionated Radiotherapy

* Median (25th and 75th percentiles)

**Comparison 4: Healthcare Systems** Patients diagnosed in a Social insurance-funded healthcare system were used as standard population. Because healthcare systems are inherently linked to geographical setting, Comparisons 3 and 4 address partially overlapping sources of variation but evaluate different exposures. The recurrence risk estimates for the Tax-funded and Mixed/Private-funded healthcare systems were calculated by standardizing the distribution of patient characteristics to that of patients diagnosed in the Social insurance–funded healthcare system. The model included WHO grade, calendar time-group, WHO classification edition, and healthcare system. Three interactions were specified: healthcare system with WHO grade, healthcare system with calendar time-group, and healthcare system with WHO classification edition.

## Results

The cohort of 4,111 patients was followed for 22,327 person-years in total, with a median follow-up of 5.2 years (range: <0.1 to 28.7 years). Of these, 3,008 had WHO-1 and 1,103 had WHO-2 meningiomas, while classification according to the 2007 and 2016 WHO editions applied to 3,217 and 894 patients, respectively. During follow-up, 645 (15.7%) patients experienced a recurrence, 276 died without recurrence, and 3,190 were censored alive and recurrence-free. Patient characteristics are shown in Table 1. Follow-up duration varied substantially between centers and calendar periods, which motivated the use of inverse probability of censoring weighting and subsequent visualization of cohort composition.

### Comparison 1: Calendar time-groups

**Fig. 1:**
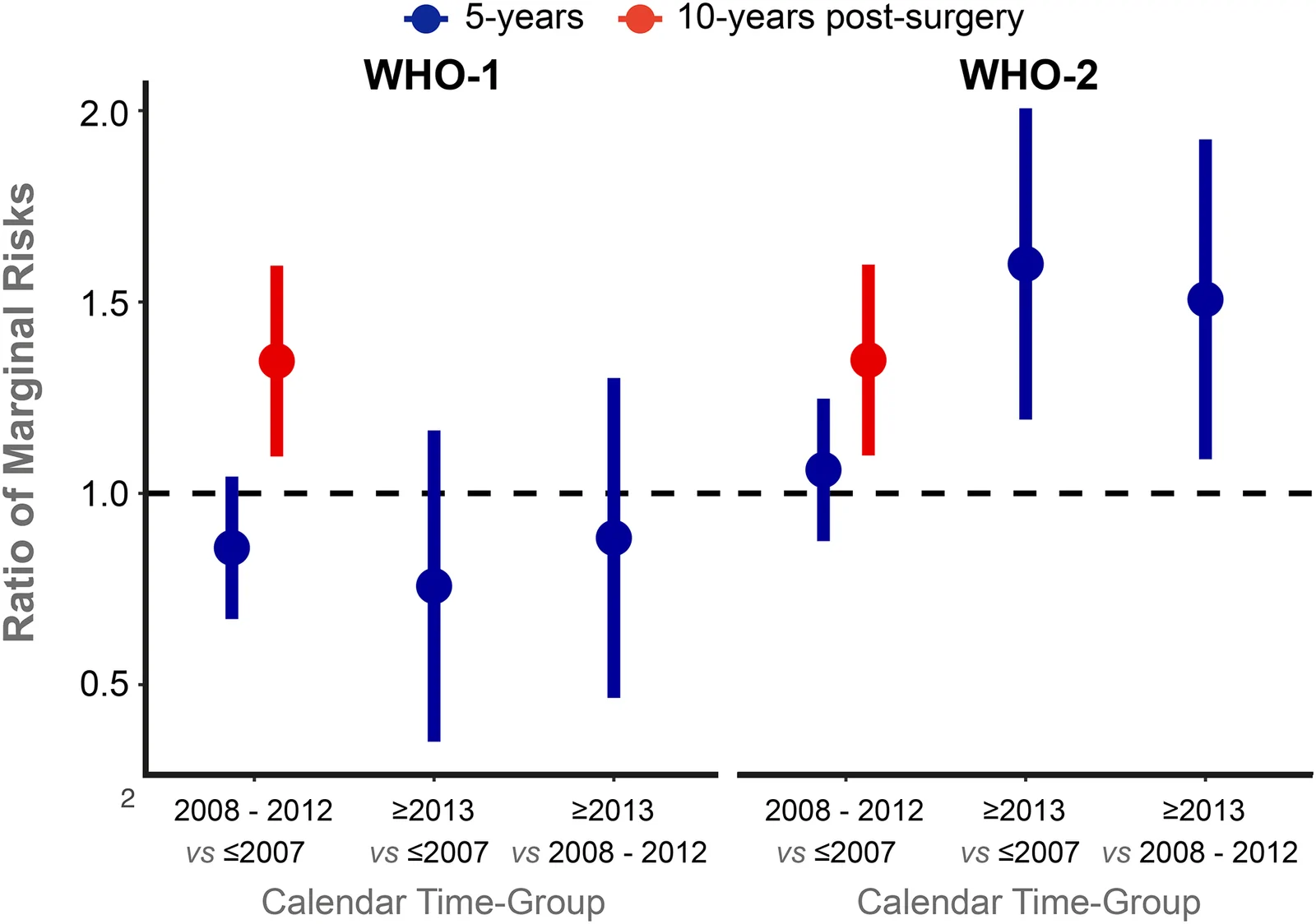
Estimates are ratios of marginal risks (averaged risks across all individuals in the population). The standard population is patients diagnosed in 2008–2012; the counterfactual marginal risks are predictions for this same population as if diagnosed in other calendar time-groups. The marginal risk for the standard population corresponds to the risk predicted by the multivariable logistic regression model. The 10-year recurrence risk was not estimated for patients diagnosed ≥ 2013, as none had yet accrued corresponding follow-up time

Patients diagnosed ≥2013 were excluded from the 10-year recurrence analysis, as none could have reached 10 years of follow-up at time of data accrual (see Supplmentary Table 1 for the unadjusted, standardized absolute risks of the standard population). A general observation was that more recent calendar time-groups were linked to higher 10-year recurrence risk estimates than earlier periods (Fig. 1). For WHO-1, no significant difference was seen in 5-year recurrence risk estimates across calendar time-groups. At 10 years, patients diagnosed in 2008–2012 showed a higher risk than if the same patients instead had been diagnosed ≤2007 (risk ratio [RR]: 1.35, 95% CI: 1.10–1.60, *P* = 0.007). For WHO-2, the predicted 5-year recurrence risk was higher in more recent calendar periods. Compared with patients diagnosed ≤ 2007, those diagnosed ≥ 2013 had a higher predicted risk (RR: 1.60, 95% CI: 1.19–2.01, *P* = 0.004), and similarly higher risk compared with those diagnosed in 2008–2012 (RR: 1.51, 95% CI: 1.09–1.92, *P* = 0.017). No significant difference was observed between the ≤ 2007 and 2008–2012 periods. At 10 years, there was a higher predicted recurrence risk among patients diagnosed in 2008–2012 compared with those diagnosed ≤ 2007 (RR: 1.35, 95% CI: 1.10–1.60, *P* = 0.006).

### Comparison 2: The WHO classification edition

**Fig. 2:**
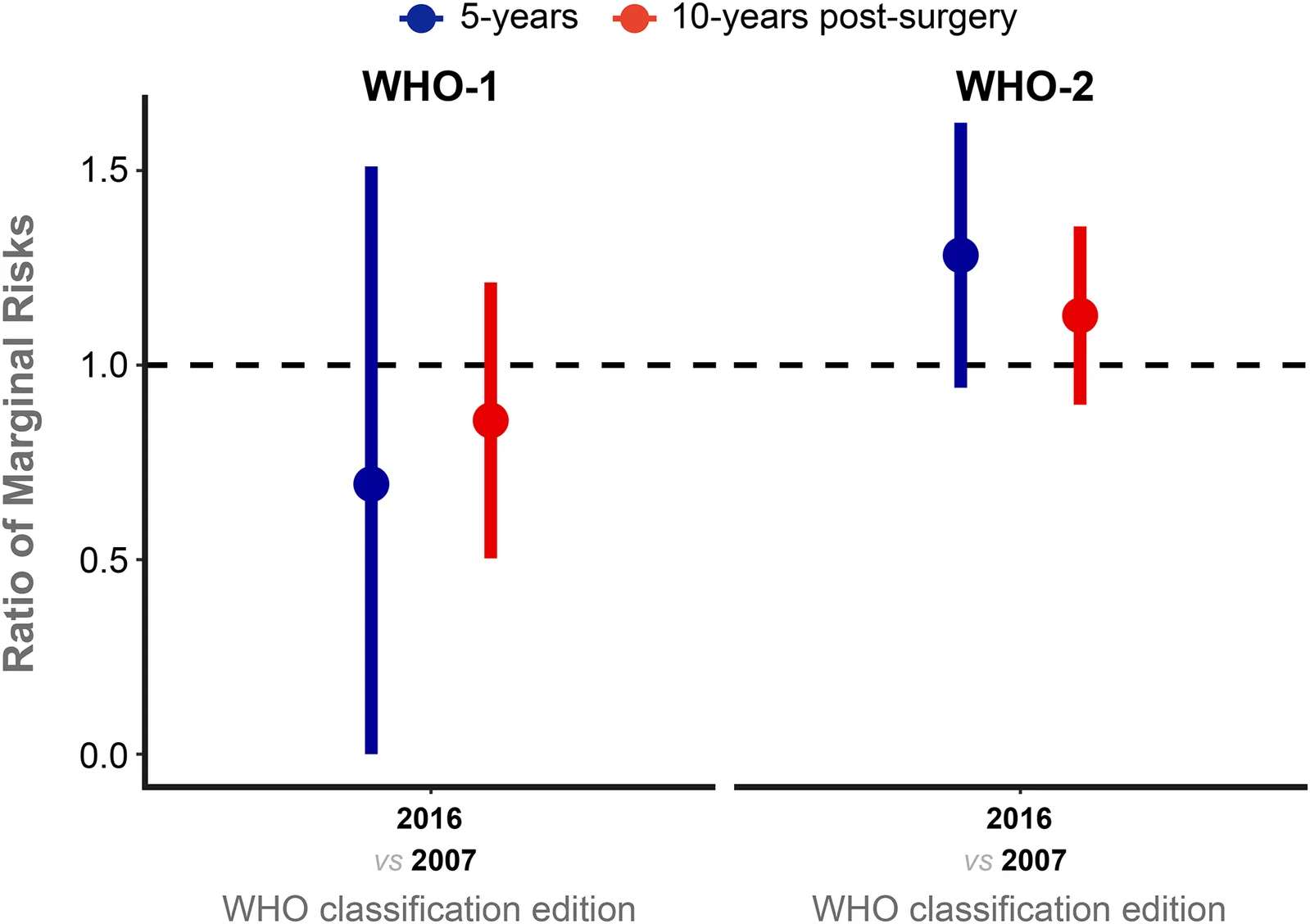
Ratios of marginal recurrence risks at 5 and 10 years post-surgery comparing WHO classifications (2007 vs. 2016), stratified by WHO grade. The standard population consists of patients classified according to the 2007 edition, with counterfactual marginal risks representing predictions for this same population as if classified under the 2016 edition

See Supplmentary Table 1 for the unadjusted, standardized absolute risks of the standard population. When comparing the 2007 and 2016 WHO classifications (Fig. 2), there was no clear evidence of a difference in recurrence risk estimates observed among WHO-1 postoperatively at 5 years (RR: 0.69, 95% CI: 0.00–1.51, *P* = 0.46), although the estimate was associated with substantial statistical uncertainty, reflecting the low number of early recurrence events. Similarly, no clear evidence of a difference was observed at 10 years (RR: 0.86, 95% CI: 0.50–1.21, *P* = 0.43). For WHO-2, a higher recurrence risk was estimated 5 years postoperatively when classified according to the 2016 edition was suggested but not statistically significant (RR: 1.28, 95% CI: 0.94–1.62, *P* = 0.10). At 10 years after surgery, the RR was 1.13 (95% CI: 0.90–1.36, *P* = 0.28) (Fig. 2).

### Comparison 3: Geographical setting

**Fig. 3:**
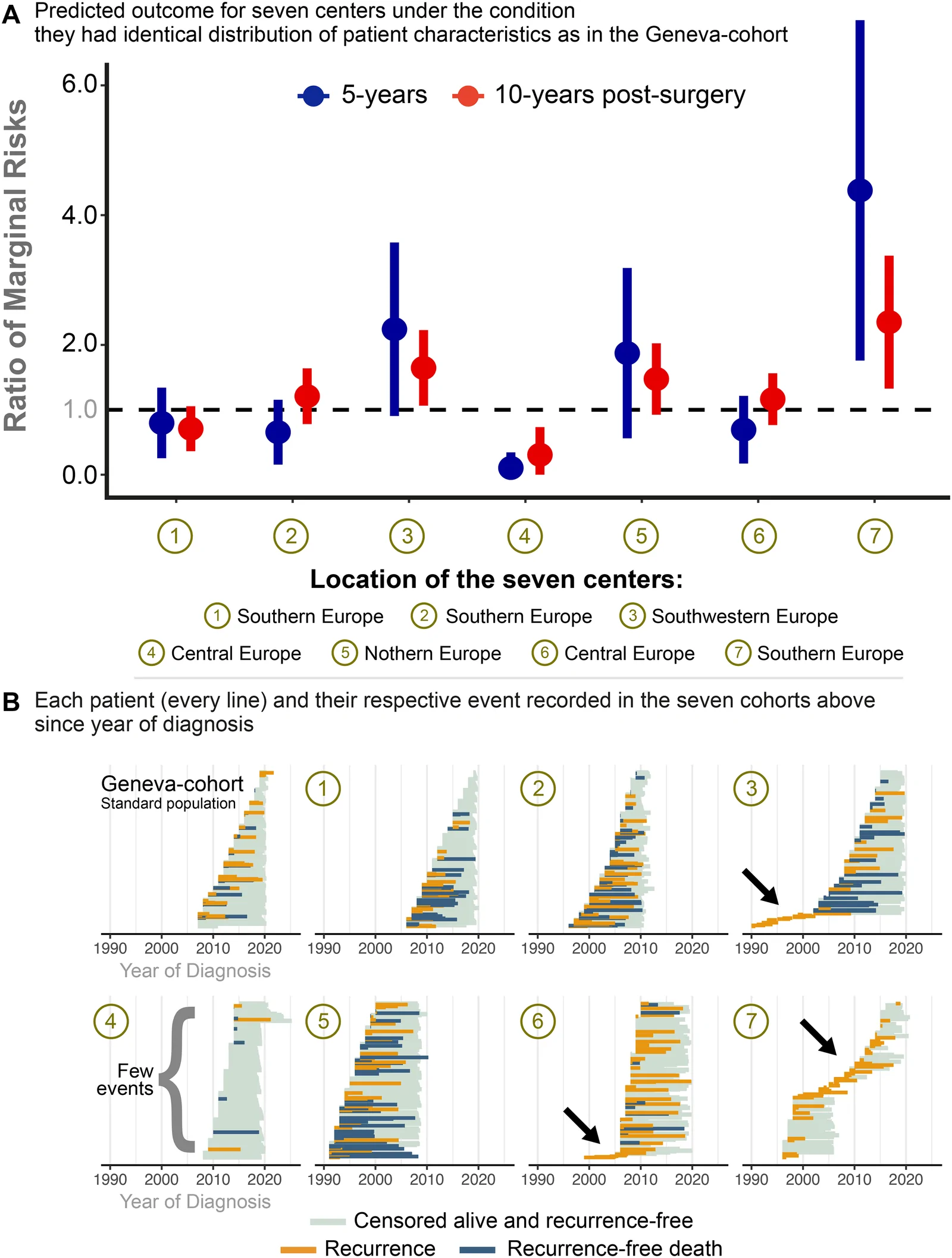
Comparison of recurrence risk across European centers with visualization of cohort data composition. (**A**) Ratio of marginal recurrence risks at 5 and 10 years post-surgery if the standard population (Geneva-cohort, Central Europe) had instead been diagnosed in seven other European centers. Only cohorts with a median follow-up of at least 5.0 years and a minimum of 20 WHO-2 patients were included. (**B**, “Cohort-Event” plots) “Cohort-Event” plots showing each patient’s follow-up from year of diagnosis to end of observation, with color denoting recurrence, recurrence-free death, or censoring alive and recurrence-free. Black arrows show irregular inclusion patterns (Center 3 and Center 7)

Using only European cohorts with a median follow-up time of at least 5.0 years and a minimum of 20 WHO-2 patients (Fig. 3A). In this scenario, the Geneva-cohort from Central Europe was used as the standard population, as it had a considerable sample size (*n* = 410) and broad representativeness across covariates, thereby serving as a reasonable sample of the other cohorts compiled (Table 1; see Supplmentary Table 1 for the unadjusted, standardized absolute risks of the standard population). When investigating the recurrence risk estimates of seven European centers, and as if the distribution of patient characteristics corresponded exactly to that of the Geneva-cohort, large fluctuations in predicted recurrence risk estimates were observed (e.g. Center 3 and Center 7, Fig. 3A). This was observed despite adjustment to key surgical, clinical, geographical, histopathological covariates, suggesting that factors beyond these parameters may influence the recurrence risk estimates - such as differences in follow-up intensity, diagnostic practices, or data recording. To further explore, each patient in the individual cohorts was visualized and followed from the year of diagnosis until end of follow-up, indicating whether the patient experienced (1) recurrence, (2) recurrence-free death, or (3) was censored alive and recurrence-free (Fig. 3B). The “Cohort-Event”-plot illustrates considerable heterogeneity in follow-up duration across centers and calendar periods, as well as variation in recurrence detection intensity. Specifically, Center 3 appeared to include patients unsystematically between 1990 and 2000 - and exclusively patients who had experienced recurrence - before transitioning to a more consecutive and systematic inclusion pattern thereafter. Similarly, Center 7 included patients consecutively during the first half of the study period, followed by a less systematic inclusion characterized by sporadic addition of patients, almost exclusively those with recurrence (most of them occurring early). This underlying data structure and recording practices likely explain why recurrence risk estimates from these centers are different and illustrate how non-uniform data accrual can influence recurrence estimates even after adjustment for key predictors, leading to heterogeneity between cohorts and impairing comparability of recurrence risk estimates across these.

### Comparison 4: Healthcare systems

**Fig. 4:**
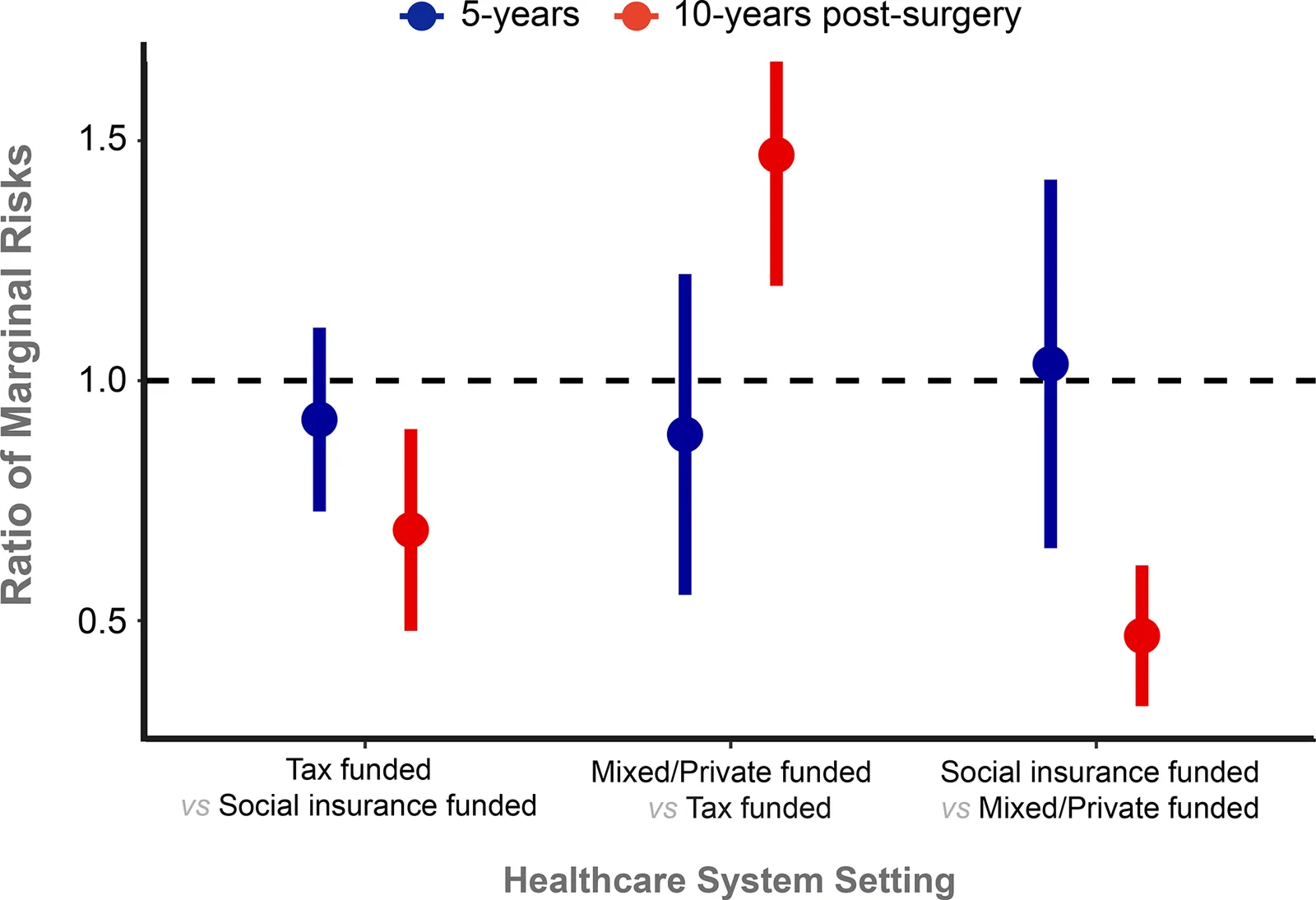
Ratios of marginal recurrence risks at 5 and 10 years post-surgery comparing different healthcare system settings. The standard population consists of patients treated within social insurance–funded systems, with counterfactual marginal risks representing comparisons to predictions for this same population as if treated in tax-funded or mixed/private-funded systems

In general, the estimates indicated differences in recurrence risk across healthcare systems (see Supplmentary Table 1 for the unadjusted, standardized absolute risks of the standard population). When applying the standard population from Social insurance–funded systems, patients were predicted to have higher 5- and 10-year recurrence risk estimates if instead diagnosed in Mixed/Private-funded systems (e.g., RR = 1.47, 95% CI 1.20–1.74, *P* < 0.001), while estimates for Tax-funded settings were generally comparable or lower (e.g., 10-year ratio = 0.69, 95% CI 0.48–0.90, *P* < 0.001) (Fig. 4). In general, the estimates suggested variation in recurrence risk estimates across healthcare systems.

## Discussion

Estimated recurrence risk was higher among patients diagnosed closer to the present time. However, recurrence in this study reflects recurrence as detected in routine practice rather than a uniformly ascertained endpoint. Differences in surveillance intensity and imaging frequency across time and institutions can affect whether - and when - recurrence is identified, and may therefore contribute to higher estimated recurrence risks in more recent periods or certain settings. Accordingly, the observed patterns should not be interpreted as evidence that treatment efficacy has worsened over time. Rather, these findings are consistent with evolving follow-up strategies with increasing imaging frequency and sensitivity.

For WHO-1, this was indicated by a higher recurrence risk estimate at 10 years but not at 5 years, likely reflecting the benign characteristics and low early recurrence rate among most of these meningiomas. The present analysis cannot extrapolate how recurrence risk estimates might have changed beyond the 10-year timeframe, although WHO-1 meningiomas are known to continue recurring thereafter [2–4, 27]. A similar pattern was observed for WHO-2, where the estimated 5-year recurrence risk was higher in more recent periods, aligning with the shorter timeframe in which recurrences are typically expected for this subgroup [28, 29]. For WHO-2, predicted 5-year recurrence risk estimate was higher under the 2016 classification edition than under the 2007 edition. The key change between these editions was the inclusion of brain invasion as a standalone criterion for WHO-2, and although prior evidence on its prognostic relevance remains mixed [30–33], this finding could suggest a short-term impact of brain invasion on recurrence risk estimates for WHO-2 patients in the *PERNS* cohort.

Among WHO-1, predicted 5- and 10-year recurrence risk estimates were similar under both the 2007 and 2016 WHO classification editions. Across healthcare systems, differences in recurrence risk estimates were observed even after adjustment for clinical, surgical, and histopathological factors [34]. These differences therefore cannot be explained by the measured variables included in the model but may still reflect unmeasured factors, including center-specific data accrual and follow-up practices. Patients treated within mixed or privately funded systems were estimated to have higher recurrence risks compared with those in social insurance–based or tax-funded systems. These findings should not be interpreted as evidence of superiority or inferiority of specific healthcare systems, but rather as an indication that system-level factors - such as follow-up routines, access to imaging, documentation practices, and financial incentives that may influence surveillance intensity and thresholds for reintervention - can influence the detection and recording of recurrence, and thereby the reported recurrence risk estimates. Among European cohorts with comparable follow-up and adequate WHO-2 representation, recurrence risk estimates varied across centers despite adjustment for clinical, surgical, and histopathological factors. Visualization of patient follow-up indicated that non-uniform data accrual and recurrence detection likely could have contributed to these differences, emphasizing how methodological heterogeneity can limit comparability across observational cohorts that otherwise appear similar in study design.

### Implications for clinical trial benchmarks

These findings also have direct implications for the interpretation of single-arm clinical trials in meningioma, where efficacy is frequently benchmarked against historical progression-free survival rates, such as PFS-6 or PFS-12 [35–37]. If recurrence detection is influenced by follow-up intensity, imaging modality, and data accrual practices - as suggested by the present analyses - then historical control estimates may not represent stable or comparable benchmarks. Consequently, observed treatment effects in such trials may partly reflect differences in outcome ascertainment rather than true therapeutic efficacy. This challenges the validity of using historical PFS-based thresholds for decision-making, including regulatory evaluation and guideline recommendations, and underscores the need for contemporaneous controls or standardized outcome assessment in future trials.

### Data transparency: towards improving comparability

Introducing “Cohort-Event” plots (like Fig. 3B) provides a transparent visualization of how individual patients contribute to each cohort over calendar time, including the timing and distribution of recurrence events, deaths, and censoring. By exposing irregularities, this tool provides insight into whether inclusion is genuinely consecutive and whether follow-up periods are adequate and uniformly applied. Incorporating such visualizations into observational studies may help identify and assess hidden biases related to non-consecutive inclusion and heterogeneous follow-up, thereby improving transparency, reproducibility, and interpretability of clinical outcome estimates and strengthening the basis for comparing recurrence risk estimates across studies.

### Perspectives

The following perspective should be regarded as hypothesis-generating, as molecular markers were not evaluated in the present study. However, while molecular classifiers are rapidly advancing in meningioma research, these appear to be partially developed with reference to outcomes reported for patient specimens collected earlier and linked to retrospective clinical follow-up. As demonstrated in the present study, recurrence estimates can vary substantially across calendar time, healthcare systems, and data recording practices. Our findings raise the hypothesis that applying modern molecular tools to historical cohorts may therefore introduce bias if the associated clinical data were accrued under heterogeneous conditions or with differing definitions of recurrence. Therefore, associations between molecular profiles and outcomes derived from historical specimens should therefore be interpreted in the context of how recurrence was detected, recorded, and followed within the original clinical setting. The recurrence and clinical outcomes remain inherently linked to the diagnostic and therapeutic practices of their original historical context - effectively “locked” in time. Ultimately, observed associations between molecular profiles and outcomes may be confounded, potentially reflecting data collection rather than true biological effects [22, 38–42].

### Limitations

A key limitation of this study is that the *PERNS* database did not apply strict inclusion or exclusion criteria, which may have introduced heterogeneity across centers in terms of patient selection, follow-up practices, and data completeness. Additionally, while regression standardization allows adjustment for measured covariates, it cannot account for unmeasured factors such as selective cohort inclusion or other center-specific data accrual practices. Furthermore, temporal changes in the assessment and recording of variables such as Ki-67 proliferation index and Simpson grade may have limited complete comparability across calendar periods despite statistical adjustment. Adjuvant fractionated radiotherapy is likely influenced by tumor aggressiveness and extent of resection and may therefore represent a downstream consequence of these characteristics rather than an independent baseline factor. However, given the observational design and the exploratory aim of comparing recurrence risk estimates under similar observed clinical conditions, adjustment for aFRT and extent of resection was considered a pragmatic approach to reduce overt differences in treatment allocation across groups. Another limitation is that meningioma classification in this study was based solely on histopathological WHO grading, without accounting for molecular markers that are now acknowledged as important prognostic indicators [15, 16]. Several genetic and epigenetic alterations were not available in the original datasets. However, implementing these would be unlikely to resolve the inconsistencies presented (since detection of recurrence is “locked” in a historical context and cannot be reevaluated according to a different scanning protocol and imaging modality). Follow-up schedules and imaging frequency were not standardized across centers and calendar periods, and detailed data on surveillance intervals were not consistently available. However, most patients in the cohort received treatment before the adoption of standardized radiological response frameworks such as the *RANO* criteria [23]. Consequently, the endpoint reflects recurrence as detected under heterogeneous surveillance practices rather than recurrence assessed under a uniform follow-up protocol.

## Conclusion

Recurrence risk estimates for meningioma varied across calendar time, WHO classification editions, geographical settings, and healthcare systems even after adjustment for key clinical and surgical factors. These findings indicate that reported recurrence risks are influenced not only by tumor biology and treatment, but also by follow-up practices and data accrual patterns. Transparent documentation of cohort composition, surveillance practices, recurrence definitions, follow-up duration, and data accrual is therefore essential to improve comparability across observational studies. Future studies should consider visualizing individual-level event timing and cohort composition to facilitate interpretation of recurrence risk estimates across settings.

## Electronic supplementary material

Below is the link to the electronic supplementary material.

Supplementary Material 1

## Data Availability

No datasets were generated or analysed during the current study.
