# Supplementary material for: Variation in meningioma recurrence risk estimates across observational cohorts: the influence of calendar time, WHO classifications, geographical settings, and healthcare systems
Source: J Neurooncol. 2026 Aug 28;179(2):60. doi: 10.1007/s11060-026-05753-7 (PMC13525048; doi:10.1007/s11060-026-05753-7)
Supplement: Supplementary file 1 [file 11060_2026_5753_MOESM1_ESM.docx]

**Supplementary table**

| **Supplementary Table 1.** The standardized absolute risk of recurrence used in the manuscript. | | | |
| --- | --- | --- | --- |
|  | **Standard Population** | **5-years standardized recurrence risk (95% CI)** | **10-years standardized recurrence risk (95% CI)** |
| **Comparison 1**  Calendar Time-Group | Diagnosed between 2008–2012 | 10.2% (8.1–12.4) | 33.6% (27.6–39.7) |
| **Comparison 2**  WHO Classification Edition | 2007 edition | **WHO-1**  10.9% (9.1–12.7)  **WHO-2**  33.3% (27.8–38.7) | **WHO-1**  27.3% (23.3–31.3)  **WHO-2**  48.1% (42.7–53.5%) |
| **Comparison 3**  Geographical Setting | Geneva Cohort | 7.1% (4.1–101.1) | 32.8% (20.5–45.0) |
| **Comparison 4**  Healthcare System | Social insurance-funded healthcare system | 14.5% (12.7–16.2) | 35.0% (29.6–40.4) |
